# Activation of LKB1 rescues 3T3-L1 adipocytes from senescence induced by Sirt1 knock-down: a pivotal role of LKB1 in cellular aging

**DOI:** 10.18632/aging.104052

**Published:** 2020-10-10

**Authors:** Fan Lan, Yan Lin, Zhenfeng Gao, Jose M. Cacicedo, Karen Weikel, Yasuo Ido

**Affiliations:** 1 The Department of Endocrinology, The First People’s Hospital in Chongqing Liangjiang New Area, Chongqing, China; 2 The Department of Laboratory Medicine, The Thirteenth People's Hospital of Chongqing, Chongqing, China; 3 The Department of Endocrinology, The First People’s Hospital in Chongqing Liangjiang New Area, Chongqing, China; 4 ALPCO Diagnostics, Salem, NH 03079, USA; 5 Bonumose LLC, Charlottesville, VA 22911, USA; 6 Boston University School of Medicine, Boston, MA 02118, USA; 7 Department of Cardiology, National Defense Medical College, Saitama, Japan

**Keywords:** Sirt1, LKB1, AMPK, adipocytes, aging

## Abstract

Previous reports have shown that excess calorie intake promotes p53 dependent senescence in mouse adipose tissues. The objective of the current study was to address the mechanism underlying this observation, i.e. adipocyte aging. Using cultured 3T3-L1 cells, we investigated the involvement of energy regulators Sirt1, AMPK, and LKB1 in senescence. Fifteen days post differentiation, Sirt1 knock-down increased senescence-associated beta-galactosidase (SA-β-Gal) staining by 20-40% (p<0.05, n=12) and both cyclin kinase inhibitor p21^Cip^ and chemokine receptor IL8Rb expression by 2-4 fold. ATP and expression of mitochondria Complex 1 were also reduced by 30% and 50%, respectively (p<0.05, n=4). Such energy depletion may have caused the observed increase in AMPK activity, despite LKB1 activity downregulation. This association between Sirt1 and LKB1 activity was confirmed *in vivo* in mouse adipose tissue. Upregulation of LKB1 activity by expression of the Sirt1-insensitive LKB1-K48R mutant in 3T3-L1 cells completely prevented the senescence-associated changes of Sirt1 knock-down. In addition, cellular senescence, which also occurs in cultured primary human aortic endothelial cells, was largely prevented by ectopic expression of LKB1. These results suggest that LKB1 plays a pivotal role in cellular senescence occurring in adipocytes and other cell types.

## INTRODUCTION

Aging is associated with or caused by a decline of cellular NAD^+^ levels [1–3]. In rodent models, restoration of NAD^+^ by supplementation with nicotinamide riboside [4] and nicotinamide mononucleotide [5] ameliorates phenotypes associated with aging including insulin resistance in high fat-fed mice [5]. Adipose tissues play a central role in insulin action and energy metabolism along with liver and skeletal muscle. Although it is known that aging can cause preadipocytes to become senescent [6], it is still not clear whether fully differentiated adipocytes undergo a similar aging phenotype that culminates with senescence. Aging is associated with declining cytosolic NAD^+^ levels, which would suppress the activity of the NAD^+^-dependent enzymes sirtuins, including Sirt1. Picard et al. [7] demonstrated that knockdown of Sirt1 in 3T3-L1 cells upregulated adipocyte differentiation. Therefore, suppression of Sirt1 may cause obesity in vivo. Resveratrol, a Sirt1 activator, was shown to prevent high-fat induced obesity in rodents [8]. Although this was not observed in human trials [9], the rodent phenomenon is consistent with this story. There is no report, however, that Sirt1 down-regulation affects adipocyte aging, or how it affects another longevity gene product, AMP-activated protein kinase (AMPK) and its upstream regulator LKB1, despite a previous suggestion [10].

The energy sensing enzyme AMPK participates in diverse arrays of signaling, including cellular senescence and aging [11]. The enzyme is heterotrimeric and is comprised of a single α, β, and γ subunit. There are two catalytic subunits, α1 and α2. AMPK activity is regulated through phosphorylation by upstream kinases (Thr172 of the α subunits is the primary activation site) and allosterically by increases in the AMP/ATP and ADP/ATP ratios [11]. It is known that α2 subunit is more sensitive to energy decline. LKB1 (STK11) is one of AMPK’s upstream kinases [11] responsible for AMPK’s activation by energy change. LKB1 possesses a nuclear localization signal and is located in the nucleus as a monomer. LKB1 is active only upon forming a heterotrimer with its cytosolically located binding partners STRAD and MO25 [12, 13]. We previously showed that Sirt1 regulates the de-acetylation of LKB1 on Ac-K48. De-acetylation of this site promotes LKB1’s subcellular nuclear to cytosolic translocation and thereby its ability to interact with its binding partners, activate and phosphorylate AMPK [14–16]. This activation cascade was later confirmed by Sinclair’s group [17]. Consistent with this scenario, AMPK phosphorylation is down-regulated when SirtT1 is knocked down [15, 18]. However, since both Sirt1 and AMPK control activation of PGC-1α, a significant factor for mitochondrial biogenesis, downregulation of both cascades likely decreases mitochondrial function [19]. Additionally, Sinclair's group showed that downregulating Sirt1 induces HIF-1α dependent but PGC-1α independent mechanisms to cause mitochondrial dysfunction [3]. Mitochondrial dysfunction decreases cellular ATP levels and increases ADP and AMP levels, which would cause AMPK activation overriding low LKB1 activity. Thus, down-regulation of Sirt1 may cause quite a complicated phenotype that could depend on the age of the cell, the cell type in which it occurs, and external factors such as the cell's environment (e.g., being in diabetic conditions). We think that LKB1 is one of the significant target molecules of Sirt1 and postulate that restoration of LKB1 activity under these conditions might ameliorate the effects of Sirt1 deficiency. In this study, we achieved this goal by expressing K48R LKB1, which is active even under Sirt1 deficiency [15].

Studying aging in adipocytes could be very challenging in vivo because of natural adipocyte turn-over that produces a mixture of young and old adipocyte populations, which makes the aging phenotype obscure. Thus, an aging study in adipose tissues is limited to preadipocytes [20]. Interestingly, Zoico and colleagues [21] found that after full differentiation, 9-10 post-induction days (PID), 3T3-L1 cells exhibited declining gene expression of genes involved in glucose metabolism, cytoskeleton maintenance, adiponectin, and increased gene expression of inflammatory cytokines, which appears to be mimicking the cellular aging phenotype. However, they did not demonstrate upregulation of senescence biomarkers. We examined this model further in this paper and found that SA-β-Gal (senescence-associated beta-galactosidase) activity is steadily increased after differentiation in a p53 dependent manner. This observation is consistent with the cellular senescence process. Thus, we used this model to investigate whether declining Sirt1 activity, by knockdown, accelerates the aging phenotype and how it relates to AMPK and LKB1 expression and activity.

Here, we found that Sirt1 knock-down further increased SA-β-Gal expression after differentiation at 15 PID, but unexpectedly AMPK expression and activity (pT172) were upregulated despite apparent downregulation of LKB1 activity. This phenomenon was accompanied by the downregulation of mitochondrial complex I expression. Pro-senescence p21^Cip^ and pro-inflammatory receptor IL8Rb expression were strongly upregulated, as was TGF-β. Lentiviral expression of LKB1-K48R, which does not require Sirt1-dependent activation, corrected all of these abnormalities including upregulation of AMPK expression and activity. Therefore, LKB1 downregulation is the main contributor to senescence induced by Sirt1 knockdown. Consistent with LKB1's role, ectopic expression of LKB1 largely prevented cellular senescence in cultured human aortic endothelial cells. Our results show a novel role of LKB1 in the aging process.

## RESULTS

### Senescence associated β-galactosidase (SA-β-Gal) was increased after differentiation and accelerated at 15 post-induction date (PID) by Sirt1 knock-down, which was prevented by LKB1 K48R expression

**Figure 1:**
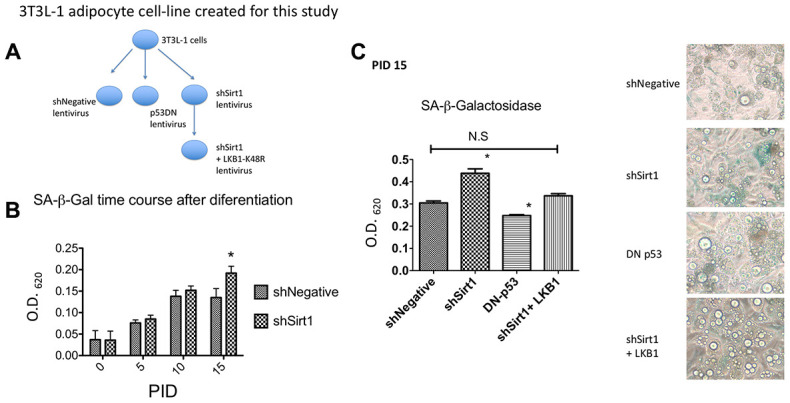
(**A**) 3T3-L1 cell lines created for this study. Using lentiviral mediated gene expression and shRNA knock-down techniques, 3T3L1 cells were infected with shNegative (non-targeted shRNA expressing) lentivirus, p53DN (dominant-negative p53 expressing) lentivirus, shSirt1 (Sirt1 targeted shRNA expressing) lentivirus, and shSirt1+LKB1-K48R (LKB1 K48R mutation) lentivirus. Infected cells were selected by corresponding antibiotics (blasticidin or hygromycin). After confirmation of lentiviral expression, these preadipocyte cell lines were induced to become adipocytes as shown in the following figures. (**B**) SA-β-Gal staining time course shNegative vs. shSirt1. Sirt1 knock-down significantly increased SA-β-Gal expression at PID 15 (15 days after starting differentiation). (*p<0.05, n=12). (**C**) SA-β-Gal staining at PID 15. Dominant-negative p53 expression decreased basal 3T3-L1 SA-β-Gal expression (*p<0.05 vs. shNegative, n=12). Expression of the Sirt1 insensitive LKB1 K48R-mutant prevented Sirt1 knock-down effects. (Not Significant to shNegative). Representative figure is shown.

In this study, we created the following 3T3-L1-based cell lines using lentiviruses: 1. shNegative expressing cells (as an infection control), 2. Dominant-negative p53 expressing cells, 3. shSirt1 expressing cells to knock-down Sirt1, and 4. shSirt1 + LKB1 K48R expressing cells (Figure 1A). The differentiation procedure was standard for 3T3-L1 cells. Many studies use differentiated cells at 8-10 PID (post induction date). However, to evaluate senescence, we examined up to 15 PID, a time-frame longer than typically used. We assessed SA-β-Gal activity by using X-gal as a substrate. In brief, formalin-fixed 3T3-L1 cells were quantified colorimetrically (Figure 1B, 1C). After the commencement of differentiation, SA-β-Gal steadily increased until it plateaued by 10 PID. There was no further increase in staining between 10 and 15 PID in shNegative cells. In general, senescence was induced by expression of p53. Lentiviral transduction of dominant-negative p53 decreased SA-β-Gal activity, corroborating the presence of cellular senescence (Figure 1C). Knock-down of Sirt1 showed a slightly higher SA-β-Gal staining in 5-10 PID which significantly increased at 15 PID (Figure 1B, 1C). In a previous paper, we demonstrated that the LKB1 K48R mutant is active without Sirt1 [15]. Thus, to determine the effect of LKB1, independent of Sirt1, we expressed the LKB1 K48R mutant using lentivirus in Sirt1 knock-down cells to determine its effects on senescence. At 15 PID, SA-β-Gal staining was no different than the shNegative control demonstrating that it suppressed or prevented the Sirt1 knock-down effect (Figure 1C).

### LKB1 activity and energy levels were influenced by Sirt1 knockdown

**Figure 2:**
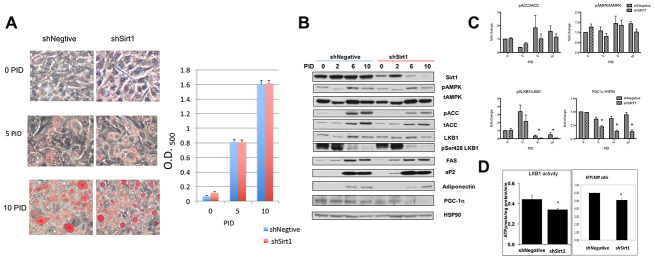
(**A**) Differentiation of 3T3-L1 cells in shNegative and Sirt1 knockdown cells. Oil O red staining image (x100). Red staining was extracted with isopropanol and absorbance was measured at 400 nm. There were no apparent effects of Sirt1 knockdown on fat accumulation. (**B**, **C**) Proteins indicating adipocyte differentiation and AMPK, LKB1, and Sirt1 signaling assessed by western blot. Sirt1 knockdown was evident at 10 PID; however, pACC/tACC and pAMPK/tAMPK showed no apparent suppression of AMPK activity. pSerLKB1/LKB1 showed suppression of LKB1 at 6 and 10 PID. A similar suppression trend was observed in PGC1α/HSP90. n=4, *p<0.05 vs shNegative. (**D**) Effect of Sirt1 knockdown on LKB1 activity and ATP/ADP ratio. LKB1 activity was assessed by LKBtide phosphorylation in immuno-precipitated samples. Sirt1 knock-down causes LKB1 activity downregulation. (n-6, *p<0.05). ATP/ADP ratios were significantly lower with Sirt1 knock-down (n=6. P<0.05). Similar experiments were repeated three times.

The effects of shSirt1 and LKB1 K48R on increasing and decreasing SA-β-gal staining, respectively, at 15 PID suggested that some drastic change may occur after the completion of differentiation (10 PID). Thus, we investigated the molecular and phenotypic changes that occur up to 10 PID, first. We assessed fat accumulation by Oil-Red O staining (Figure 2A) along with pAMPK (pT172-AMPK), pACC (AMPK substrate), total-AMPK, total-ACC, and LKB1 expression and activity (Figure 2B, 2C). Full fat accumulation was observed at 10 PID. Colorimetric analysis of oil-red staining showed no difference in fat accumulation between shNegative- and shSirt1-infected cells. We assessed insulin-stimulated glucose uptake and isoproterenol stimulated glycerol release and found no effect of Sirt1 knock-down (data not shown). Adipocyte differentiation markers FAS, aP2, and adiponectin became evident at 6-10 PID (Figure 2B). In shSirt1 cells, Sirt1 expression was 80-90% reduced at 6-10 PID, but there were no remarkable changes in these differentiation markers, supporting the Oil-Red O staining results.

During differentiation, pACC and tACC levels were increased (6-10 PID), but these were not affected by Sirt1 knock-down (Figure 2B, 2C). Since there were no significant changes in the pACC to tACC ratio, we concluded that active (un-phosphorylated) ACC was increased. ACC is an enzyme responsible for fatty acid synthesis, therefore this increase is reasonable. Overall, there was no remarkable change in pAMPK, tAMPK, or LKB1 levels during differentiation or by Sirt1 knock-down (Figure 2B). pSer428-LKB1 was strongly increased during 0-4 PID and subsequently decreased (Figure 2B, 2C). As explained in Methods, this site is phosphorylated by PKA or RSK, which are activated by phosphodiesterase inhibitor IBMX and insulin, agents used for induction of differentiation during this period. Therefore, this observation was not surprising. pSer428-LKB1/LKB1 ratio was consistently decreased after (Figure 2C). Remarkable decreases were observed in PGC-1α levels in 6-10 PID in shSirt1 cells. In a separate set of experiments with cells at 10 PID, we assessed LKB1 activity *in vitro* using LKBtide as a substrate and cellular ATP/ADP ratios (Figure 2D). LKB1 activity and the ATP/ADP ratio were decreased 20-30% in shSirt1 cells, suggesting that although the cells looked normal, cellular dysfunctions were being caused by Sirt1 knock-down. Decreased PGC-1α is likely responsible for the decreased energy levels through impaired mitochondrial biogenesis.

The results showed decreased LKB1 activity simultaneously occurring with low energy levels which may work to inhibit and/or activate AMPK, thus, potentially generating a neutral effect. We further investigated pAMPK levels in cells at 8-9 PID. In 2 out of 4 experiments, pAMPK was reduced, but in the other 2 experiments, no apparent change was observed. Thus, consistent with our previous report, LKB1 activity was reduced by Sirt1 knock-down, but pAMPK levels fluctuated and were inconsistently affected in differentiated 3T3-L1 cells in this intermediate PID timeframe.

### Increased cellular senescence by Sirt1 knock-down at 15 PID was associated with strong increases in AMPK and pT172 AMPK expression, which were prevented by LKB1-K48R expression

**Figure 3:**
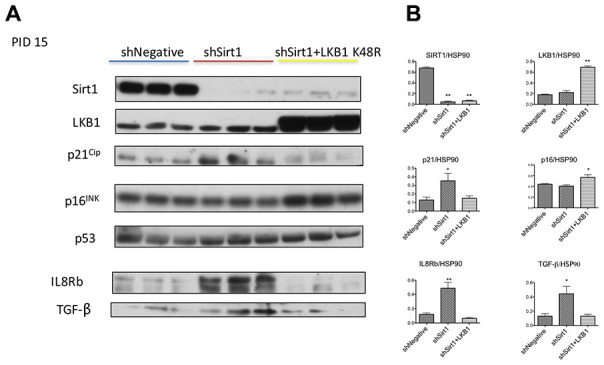
(**A**, **B**) Western blots of proteins related to senescence and inflammation at PID 15 in shNegative, shSirt1, and shSirt1+LKB1 K48R cells. (**A**) The aforementioned proteins were assessed by western blot in PID 15 shNegative, Sirt1 knockdown, and Sirt1 knockdown + LKB1 K48R expressing 3T3-L1 cells. Upregulation of cyclin kinase inhibitor p21^CIP^, inflammatory chemokine receptor IL8b, and TGF-beta was observed in Sirt1 knockdown cells, and LKB1 K48R prevented these changes. (n=4, *p<0.05 vs. shNegative).

Firstly, we confirmed senescence using markers other than SA-β-Gal**.** In general, cellular senescence occurs by activation of p53-p21^Cip^ or p16^INK^ signaling. We observed increased expression of p21^CIP^ by Sirt1 knockdown (Figure 3A, 3B, shNegative vs. shSirt1). In 3T3-L1 cells, basal level expression of p16^INK^ was already noted and not influenced by Sirt1 knock-down (Figure 3A, 3B). This continuous expression of p16^INK^ was reported before [26, 27], and the precise roles of the expression are not clear. Cellular senescence is associated with and/or caused by various pro-inflammatory cytokine signaling pathways. These cytokines bind to the CXCR2 (IL8Rb) receptor to exert their inflammatory signaling to cause cellular senescence [28]. We analyzed this protein expression by western blot and found it highly upregulated (Figure 3A, 3B). Because of this result, we checked 10 PID sample and found that CXCR2 (IL8b) expression was already high at this stage (data not shown). Similarly, TGF-β expression was increased (Figure 3A, 3B). LKB1 K48R expression prevented or reversed the upregulation of p21^Cip^, IL8Rb, and TGF-β (Figure 3A, 3B). It also upregulated P16^INK^ expression modestly (Figure 3A, 3B). Collectively, Sirt1 knock-down 3T3-L1 cells at 15 PID demonstrated the biochemical signatures consistent with cellular senescence and these were prevented by LKB1-K48R expression.

**Figure 4:**
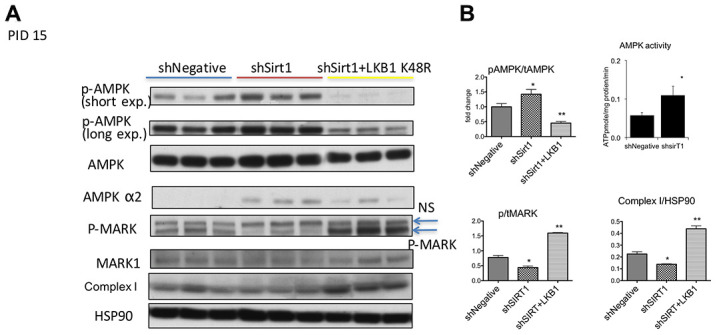
(**A**, **B**) Western blots of proteins related to and AMPK, LKB1, and Sirt1 signaling and mitochondrial dysfunction at PID 15 in shNegative, shSirt1, and shSirt1+LKB1 K48R cells. p-T172 AMPK and AMPK alpha2 were increased despite decreased p-MARK (LKB1 substrate) in Sirt1 knockdown cells. LKB1 K48R increased p-MARK and decreased p-T172 AMPK. It also significantly upregulated mitochondrial complex I proteins. The ratio of phospho-to-total MARK: A decrease in this ratio indicates a decrease in LKB1 activity caused by Sirt1 knockdown and upregulated by LKB1 K48R expression. Complex 1 expression normalized by HSP90: As seen in (**A**), expression of LKB1 K48R not only ameliorates LKB1 activity, it also increases mitochondrial complex I expression. (n=4, *p<0.05 vs shNegative). AMPK activity was increased by shSirt1 cells: AMPK activity was assessed by using ^32^P-ATP in vitro in the SAMS peptide assay. (*p<0.05 vs shNegative, n=6).

We next assessed AMPK signaling at 15 PID (Figure 4A, 4B). We found consistent robust T172 phosphorylation of AMPK by shSirt1 in all repetitions of this experiment. This unexpected result was also confirmed in a separate experiment by *in vitro* activity assay (Figure 4B). We also observed a slight increase of total AMPK, and also an increased expression of the AMPK α2 subunit (Figure 4A). The LKB1 substrate MARK1 protein phosphorylation was decreased, suggesting downregulation of LKB1 activity, consistent with LKB1 activity downregulation observed at 10 PID. Since TGF-β and inflammation may activate TAK1, which is another AMPK kinase, we assessed TAK1 phosphorylation. TAK1 expression was barely detectable in 3T3-L1 cells, and no detectable signal was observed with phospho-antibodies (data not shown). Similarly, we also could not detect a reliable band of CamKK by western blot. We assessed mitochondria oxidative phosphorylation complex expression and found downregulation of complex I in Sirt1 knock-down cells (Figure 4A, 4B). Collectively, at 15 PID, Sirt1 knock-down downregulated LKB1 activity, but AMPK signaling was upregulated likely due to low energy conditions and AMPK alpha2 overexpression. Interestingly, LKB1 K45R expression strongly suppressed pT172-AMPK, while a 2-fold increase in phospho-to-total MARK1 ratio, which is indicative of LKB1 activation (Figure 4A, 4B), was observed. This LKB1 activation was accompanied by a 2-fold increase in Complex I expression (Figure 4A, 4B).

### Modulation of LKB1 acetylation and activation occurs in mouse fat tissues

**Figure 5:**
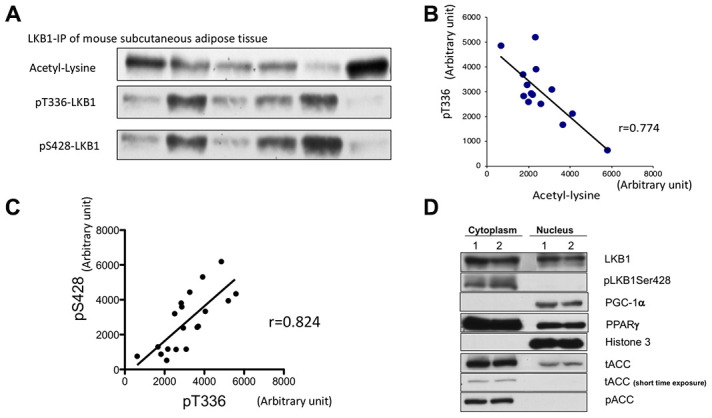
**Assessment of LKB1 acetylation and phosphorylation in mouse adipose tissue.** (**A**) Western blot of LKB1 post-translational modifications: acetyl-lysine, pS428, and pT336. LKB1 from mouse subcutaneous adipose tissues were immunoprecipitated, and LKB1 acetylation, pS428, and autophosphorylation site pT336 were assessed by western blotting. (**B**) LKB1 acetylation and pT366. The degree of acetylation of LKB1 is negatively correlated with pT336 (r=0.77, p<0.05), suggesting that LKB1 acetylation, which is regulated by Sirt1 activity, affects LKB1 activity. (**C**) Correlation between S428 and pT336. pS428 and pT336 were positively correlated (r= 0.824 p<0.05), suggesting that pS428 indicates activation (**D**). pS428 LKB1 is located in the cytosol. Two sets of samples were fractionated. LKB1 pSer428 was only observed in the cytosolic fraction, suggesting that LKB1 pSer428 is indicative of active (cytosolic) LKB1 content. Similar experiments were repeated three times.

We examined whether suppression and activation of LKB1 via acetylation can occur in adipose tissue of the mouse *in vivo*. Previously we demonstrated that knock-down of Sirt1 increases LKB1 acetylation while Sirt1 overexpression and activation by resveratrol decreases acetylation [15]. Therefore, the acetylation status of LKB1 is a likely indicator of Sirt1 activity. In vivo, Sirt1 activity can be modulated by circadian rhythm and feeding-fasting cycle, thus, we expected that LKB1 acetylation may fluctuate. LKB1 was immunoprecipitated from mouse subcutaneous adipose tissues and blotted with acetyl-lysine, pS428, and pT336, a primary auto-phosphorylation site (Figure 5A). Phosphorylation of T336 LKB1 only occurs when LKB1 forms the active complex with STRAD and MO25, thus, is an indicator of LKB1 activation. We found that LKB1 acetylation was negatively correlated with pT336 (Figure 5A, 5B). In addition, there was a strong positive correlation between pT336 and pS428 (Figure 5A, 5C). The results suggest that Sirt1 activity may control LKB1 activity *in vivo* and this can be monitored by assessing pSer428 LKB1. We previously demonstrated such a relationship in aortic endothelium after acute exercise [29].

We then characterized how pSer428-LKB1 may be indicative of LKB1 activity (Figure 5D). In 3T3-L1 cells at 7 PID, we separated cytosolic and nuclear fractions and blotted with pS428-LKB1 and LKB1. While LKB1 presented in both fractions, pS428-LKB1 was only observed in the cytosol. This result was largely consistent with a previous report [30]. Since LKB1 active complex localized only in cytosol, p428-LKB1 appears to reflect the amount of the active complex unless upstream kinase activation is present.

### Sirt1 knock-down induced LKB1 suppression and energy depletion occurred in other cell types

**Figure 6:**
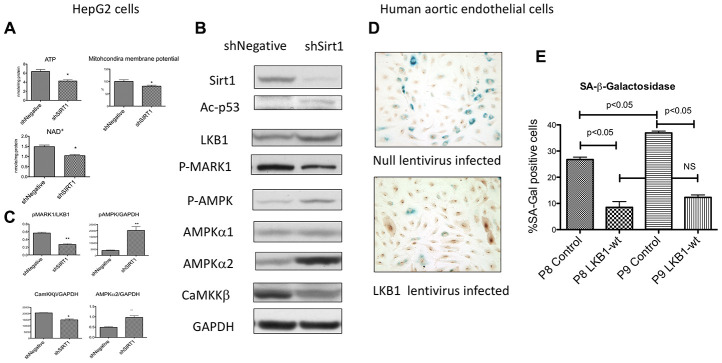
(**A**–**C**) **Effects of Sirt1 knock-down in HepG2 cells after 3-5 passages.** (**A**) Sirt1 knock-down decreased ATP, mitochondria membrane potential, and NAD^+^ levels (n=6, *p<0.05). (**B**, **C**) Sirt1 knock-down increased pAMPK levels and AMPK α2. It decreased LKB1 specific activity (pMARK1/LKB1) and CaMKKβ. (n=4, *p<0.05). (**D**, **E**) Effects of LKB1 epitopic expression by lentivirus on cellular senescence of cultured primary human aortic endothelial cells. LKB1 or null lentivirus was infected to the endothelial cells at passage 5 and passaged up 9. SA-β-Gal activity by X-gal staining and LKB1 immunostaining (DAB as substrate) were performed at passages 8 and 9. A significant increase in the percentage of SA-β-Gal positive cells was observed between passages 8 and 9 in null-lentivirus infected cells (n=6, *p<0.05). LKB1 expressed cells showed a significantly less percentage of positive cells at both passages and had no increase between passages 8 and 9.

We examined other cell lines to determine whether the phenomena observed above, such as a decreased ATP, suppression of LKB1 activity, and activation of AMPK by Sirt1 knock-down, are unique to differentiated 3T3- L1 cells. We reported previously that acute suppression of Sirt1 by lentiviral shRNA decreased AMPK activity in un-passaged HEK293T cells and HepG2 cells [15, 18]. We hypothesized that modulation of AMPK activity may depend upon the duration of Sirt1 suppression. Therefore, we examined several cells types in which we suppressed Sirt1 with shSirt1 through several passages. Decreased ATP levels were observed in 293T cells and HepG2 cells (Figure 6A) at 3-5 passages after Sirt1 knock-down. In HepG2 cells, we also observed a decreased mitochondrial membrane potential (6A), suggesting that the ATP defects were mediated through mitochondrial dysfunction. Cellular NAD^+^ levels were also decreased (6A). In HepG2 cells at 3-5 passages after Sirt1 knock-down, total LKB1 levels were increased (Figure 6B), but activity was decreased as shown by downregulated p-MARK1 (Figure 6B, 6C). In a separate set of experiments pSer428-LKB1 was decreased in Sirt1 knock-down (data not shown). Despite decreased LKB1, AMPKα2 and p-T172 AMPK were increased (Figure 6B, 6C). In HepG2 cells, CamKKβ (another upstream kinase of AMPK) expression was decreased by Sirt1 knock-down. Similarly, mitochondrial Complex I downregulation was also observed (data not shown). We also observed upregulation of AMPKα2 in human aortic endothelial cells by Sirt1 knock-down (data not shown). However, we did not observe these effects in the INS-1 β-cell line. These results attest that Sirt1 knockdown effects can be seen in other cell types, but not all cell types, after longer suppression.

### Ectopic expression of LKB1 prevented cellular senescence in human aortic endothelial cells

Finally, we examined whether ectopic expression of LKB1 slows cellular senescence in primary human cells. For years we have used human primary endothelial for studying diabetic complications and know that human aortic endothelial cells (HAEC) from adult donors become senescent relatively quickly, typically within less than 10 passages even under normal culture conditions. We examined whether ectopic expression of LKB1 affects SA-β-Gal expression. Passage 5 HAEC were infected with lentivirus expressing wild-type LKB1 or a null-expression control virus. There was no effects on cellular proliferation. The cells at passage 8 and 9 were examined for SA-β-Gal staining together with LKB1 immunostaining. The percentage of SA-β-Gal positive cells was increased from passage 8 to 9 in control cells (Figure 6D, 6E). The percentage of SA-β-Gal positive cells in LKB1-lentivirus infected cells were significantly lower than in control cells at passages 8 and 9 and no significant increase was observed between passages 8 and 9 (Figure 6D, 6E). This result showed that LKB1 plays a pivotal role in cellular aging in primary human cells as well as differentiated 3T3-L1 cells.

## DISCUSSION

This study demonstrated that 3T3-L1 cells undergo an "aging" phenotype after full differentiation, and this was augmented by Sirt1 knock-down after full differentiation. The process was accompanied by mitochondrial dysfunction characterized by reduced expression of Complex I and upregulation of inflammatory cascade proteins. Increased expression of AMPK α2 and p-T172 AMPK, and AMPK enzyme activity were observed despite apparent suppression of LKB1 activity. This downregulation of LKB1 plays significant roles in the process since the expression of LKB1 K48R completely prevented these phenotypes and signal transduction abnormalities. This result attests that: 1. one of the significant targets of Sirt1 could be LKB1, 2. AMPK activation did not prevent senescence, and 3. LKB1 activity seems to be required to maintain mitochondrial function independent of Sirt1 and AMPK. Furthermore, dysregulation of Sirt1-controlled LKB1 acetylation and activity may cause *in vivo* aging in fat cells.

LKB1 has a nuclear localization signal at its N-terminus [13], which is responsible for constitutive nuclear localization. On the other hand, LKB1's protein binding partners STRAD and MO25 that are required for LKB1 kinase activity are located in the cytosol [12, 13]. Therefore, LKB1 is a nucleus-cytosol shuttling protein. Interestingly, Sirt1 also shuttles between the nucleus and cytosol [27]. In adipocytes, it was demonstrated that in addition to Sirt1 [7], cytosolic Sirt2 also participates in differentiation processes presumably by moving to the nucleus [28]. Thus, multiple sirtuin family members appear to control fat differentiation [29]. This may be a part of the reason why we did not observe the apparent promotion of adiposity by Sirt1 down-regulation. In a previous publication in which we demonstrated the regulation of LKB1 by Sirt1, we found that LKB1 did not bind to Sirt2 or Sirt3. Thus, we think LKB1 de-acetylation and activation are preferentially regulated by Sirt1.

AMPK is recognized as a longevity gene [30]. Pharmacological activation of AMPK by metformin may extend life-span [31]. We previously demonstrated that activation of AMPK prevented stress-induced cellular senescence in human primary keratinocytes [32, 33]. Similar findings to prevent aging by activation of AMPK were reported by many other researchers [34–37]. In this study, contrary to such observations, we found that AMPK activation did not prevent senescence. Instead, strong AMPK activation was coincident with induction of a senescence phenotype. In the literature, there are also a number of papers suggesting the activation of AMPK promotes senescence [38–43]. The first report came in 2003 in the study of replicative senescence in human IDH4, IMR-90, and WI-38 fibroblasts [38]. During senescence, increased cellular AMP: ATP ratio and AMPK activation were observed, and AMPK activation appeared to accelerate senescence by reducing the expression of the RNA-binding protein HuR and the expression of p16 [38]. Thus, regarding cellular senescence or maybe aging in general, there is literature claiming the dichotomous effects of AMPK activation.

Mechanistically, phosphorylation and activation of p53 by AMPK leading to increased p21^CIP^ has been proposed for G1 cell cycle arrest and senescence reported firstly in early 2000 [44–48]. At that time, when we read these papers, we tested whether continuous activation of AMPK by AICAR (5-Aminoimidazole-4-carboxamide ribonucleotide) induces cellular senescence using human umbilical vein endothelial cells. AICAR was found to cause G1 arrest as reported by others, but the effect was immediately reversed once AICAR was removed from the cells’ media, suggesting that the AMPK activation per se does not cause senescence. In addition, we also reported that AMPK activation did not increase p53 phosphorylation in various types of human primary cells [32]. Taken together, AMPK activation could work to prevent or promote cellular senescence in a context-dependent manner.

**Figure 7:**
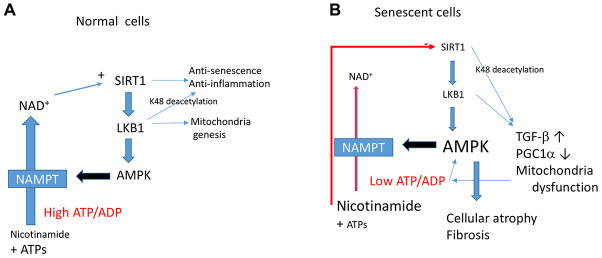
The Sirt1-LKB1-AMPK cascade in normal (**A**) vs. senescent cells (**B**). In senescent cells, a continuous decrease in ATP/ADP ratio leads to activation of AMPK. This leads to mitochondrial dysfunction induced by Sirt1-LKB1 down-regulation through a decreased PGC1α. Low ATP/ADP ratio decreases NAD^+^ production and induces Sirt1 inhibition. Continuous activation of AMPK leads to aging phenotypes such as cellular or tissue atrophy and fibrosis.

So, what are the factor(s) determining senescence-promoting effects of AMPK? Although we do not have clear answers, this study gives us some hints. AMPK is known to regulate energy homeostasis, to maintain ATP levels under stress conditions [11]. However, cells that undergo senescence show lower energy status as shown by others [38] and by us in this study. Thus, AMPK activation during the senescence process fails to maintain energy levels. AMPK activation upregulates catabolic processes to produce more ATP [11]. Increased expression of NAMPT (nicotinamide phosphoribosyltransferase) by AMPK, which synthesizes NAD^+^ from nicotinamide, could be one of such cascades [49]. This leads to increasing NAD^+^, which activates sirtuins [49], including Sirt1. Thus, in normal cells, the AMPK-NAMPT-Sirt1 cascade activates PGC-1α for mitochondrial biogenesis to restore energy levels (Figure 7A). It was demonstrated in hepatocytes that cytosolic NAD^+^ levels are controlled by ATP levels [50]. Thus, if ATP levels are continuously low, NAD^+^ levels are also low, and in such case, no activation of sirtuins would occur despite activation of AMPK. Since activation of AMPK inhibits protein synthesis [51], this induces cellular or tissue atrophy as observed in aged tissues. Sirt1 inhibition by accumulation of nicotinamide likely accelerates this process giving rise to the aging phenotype. This would lead to the downregulation of LKB1 activity (Figure 7B), as shown in this study, which is consistent with previous observations [15, 17]. We found that activation of LKB1 by LKB1 K48R reverses the effects. Thus, LKB1 activation may be a critical point. LKB1, directly and indirectly, controls many molecules by phosphorylation [13]. AMPK-independent effects of LKB1 on energy metabolism, including mitochondrial biogenesis, are less well known, but there are a few examples. Schwann cell-specific LKB1 knock-out mice revealed declining NAD^+^, ATP, and axonal degeneration that resembles diabetic neuropathy despite activation AMPK [52]. In that paper, double knock-out of AMPK α1&2 subunits did not show the same phenotype, confirming an AMPK independent effect. Similarly, LKB1 deficient hematopoietic stem cells showed mitochondrial defects and decreased ATP levels [53–55]. These phenomena were not observed in AMPK α subunits double knock-out mice [55], suggesting AMPK independent effects. Thus, there is a link between LKB1 and mitochondrial function independent of AMPK, and this issue is worth further investigation.

Some may question how p-AMPK can be increased while LKB1 is suppressed. In a study using LKB1 hypomorphic mice, which express only 10-20% of the LKB1 expressed in wild-type mice, phenformin-mediated AMPK activation in adipocytes was as high as in wild-type mice, and there was no difference in increased pACC/ACC levels [56]. This attests that unlike other AMPK-related kinases, energy levels determine p-AMPK levels rather than LKB1 amount or activity. There is also a possibility that CaMKKβ activity is increased; however, we could not see a reliable band because of low expression as reported by Gormand et al. [56]. They also found that Sirt1 knock-down decreased rather than increased CaMKKβ expression. Thus, CaMKK mediated activation seems to be unlikely. Another question: why does K48R LKB1 fail to increase pAMPK, as shown in Figure 4A? We found that K48R LKB1 increases LKB1 activity as assessed by activity assay and direct substrate phosphorylation in a previous study [15]. In this study, there is a discrepancy between Mark-1 phosphorylation, which is an energy level insensitive substrate of LKB1, vs AMPK phosphorylation, which is an energy level sensitive substrate of LKB1. Therefore, the most plausible explanation is that pAMPK is suppressed due to the restoration of energy levels.

Finally, we found in this study, a possible link between LKB1 and AMPK α2 subunit. This effect was observed in many other cell types, and therefore, there must be molecular connection. Currently, the mechanism of this action is unknown. LKB1 K48R expression effectively prevents this phenomenon, so it is likely to be related to LKB1 activity. It is known that the E2F1 transcription factor positively controls the expression of AMPK alpha 2 [57]. Thus, it may be related to E2F1, but the link to Sirt1 and LKB1 is not clear. This outstanding question remains to be determined in future studies.

In conclusion, we demonstrated that when Sirt1 activity is compromised, activation of LKB1 but not AMPK activation may ameliorate cellular dysfunction. This AMPK independent LKB1 function is worthy of further study *in vivo*.

## MATERIALS AND METHODS

### Cell culture

NIH3T3-L1 (ATCC CL-173), HepG2 (ATCC HB-8065) and HEK293T (ATCC CRL-11268) were purchased from ATCC (American Type Culture Collection, USA). The cells were cultured in appropriate media recommended by ATCC. NIH 3T3-L1 cells were cultured in DMEM with high glucose containing 10% bovine serum, and HepG2 cells were with MEM with low glucose containing 10% FBS. HEK293T cells were used for making lentivirus and propagated with Opti-MEM containing 5% FBS. Human aortic endothelial cells were obtained from Lonza (USA) and cultured using EGM-2 medium following their recommendations. The cells were passaged at 70% confluency.

### Sub-cloning of NIH3T3-L1 cells

Newly purchased NIH3T3-L1 cells were expanded and frozen in liquid nitrogen. This initial stock was very resistant to adipogenic differentiation with the standard 3T3-L1 differentiation protocol which was also reported by other [58]. Because of this, sub-cloning was performed by limited dilution in 96 well plates. Clones made from single cells were expanded and tested for their ability to differentiate. Those that differentiated well were expanded; stocks were then cryopreserved and used for the studies herein.

### Differentiation of NIH3T3-L1 cell to adipocytes

3T3-L1 cells were grown in DMEM 10% calf serum which was changed every 2 days. To start differentiation, the cells were re-plated in 12 well dishes. Two days after post confluence, the cells were incubated with DMEM 10% FBS supplemented with 0.5 mM IBMX, 1 μM dexamethasone, and 1 μg/ml insulin for 2 days (0-1 PID) followed by 2 days with 1 μg/ml insulin only (2-3 PID). The cells were then maintained DMEM containing 10% FBS medium (4-15 PID). Consistently, approximately 70% of cells became fat droplet containing cells.

### Oil Red O staining

Oil Red O staining was performed on formalin-fixed cells using a protocol described here; http://www.ihcworld.com/_protocols/special_stains/oil_red_o_ellis.htm.

### Senescence associated β-galactosidase (SA-β-Gal) assay

The assay was performed as previously published [32]. In brief, after fixation with 2% formaldehyde and extensive wash, the cells were incubated with SA-β-Gal staining solution overnight. After observation under a microscope, the blue color products were dissolved with DMSO and absorbance measured at 620 nm.

### Western blotting

Western blots were performed as previously described [15]. Equal amounts of proteins were loaded. Antibodies used this study were from Cell Signaling Technologies (USA); AMPK (5832), pT172-AMPK (50081), acetyl-CoA carboxylase (ACC, 3676), pS79-ACC (11818), p-MARK (4836), MARK1 (3319), from Santa Cruz Biotechnology (USA); LKB1 (sc-32245), p-S428LKB1 (sc-271924), Sirt1 (sc-15404), from Merck (USA); Sirt1 (07-131), from Abcam (USA); FABP4 (ab23693), OXPHOS (ab110413), AMPK alpha2 (ab3760). CamKK β antibody was a gift from D. Grahame Hardie (Dundee, UK).

### Construction of recombinant lentiviruses: shRNA for human Sirt1 or mouse Sirt1, and dominant-negative p53, and LKB1K48R or LKB1 wt

The lentiviral shRNAs targeting human Sirt1 and its negative control were previously made and fully described in [15]. We employed the same procedure to make a lentiviral shRNA for mouse Sirt1. For mus musculus Sirt1 mRNA (NM_019812) we used the sequence 5'-gacccaagaccattcttca-3' as the shRNA target. The dominant-negative p53 expressing lentivirus was created as previously described [32]. Human LKB1 and LKB1 K48R mutant were prepared as described before [15]. Lentivirus vectors expressing LKB1s were created using Lenti6 gateway donor vector (Invitrogen, USA) by LR reaction.

### Determination of cellular ATP and ADP

Cellular ATP and ADP were determined by luminometric assay as described in [16].

### LKB1 analysis of mouse subcutaneous adipose tissue

Eight-week-old C57BL/6J mice (both male and female) were purchased from Jackson Labs (Bar Harbor, ME), fed a standard chow diet, and kept in standard housing with a 12hr/12hr light and dark cycle. Standard care was taken according to the Boston University IACUC guidelines. The mice were euthanized with CO_2_ gas. The subcutaneous adipose tissue was removed and frozen in liquid nitrogen. Fat tissues were homogenized with lysis buffer and LKB1 was immunoprecipitated with a protein G-anti-LKB1-IgG conjugated complex as prev iously described [15]. LKB1 was eluted and subjected to SDS-PAGE and western blotting.

### Assessment of AMPK and LKB1 activity

Activation of AMPK requires phosphorylation at Thr172 in its catalytic subunit (mouse α1 sequence), and the antibody detecting this site is widely used for monitoring activation. However, since other phosphorylation sites in the catalytic subunit such as Ser485 may inhibit activity, we also assessed enzyme activity using SAMS peptide *in vitro* [59]. Kinase active LKB1 is a heterotrimetric complex containing LKB1, STRAD, and MO25. The enzyme activity can be assessed *in vitro* using LKB1tide as a substrate with ^32^P-ATP [15]. There are 4 auto-phosphorylation sites including Thr336. Previously, we purchased pThr336 antibody and used it in this study [15]. This antibody was only useful in immunoprecipitation or exogenously expressed samples and is not commercially available anymore. pSer428 (mouse Ser431) antibody is widely available and easily detectable in whole cell extract. Although this phosphorylation site is frequently used for monitoring activation, it is known to be phosphorylated by PKA (cyclic dependent protein kinase) and RSK (ribosomal S6 kinase) [60]. Therefore, usage of this antibody may require some caution, particularly in the presence of activators (such as IBMX or insulin). We evaluated this antibody along with pThr336 antibody to assess activity. MARK1 is a substrate protein of LKB1 and pMARK1 antibody is commercially available. The ratio of pMARK1/total MARK1 was used for an indicator of LKB1 activity [15]. Since MARK1 protein expression is usualy constant, the ratio pMARK1/LKB1 may indicate a specific activity of LKB1.

## References

[1] Massudi H, Grant R, Braidy N, Guest J, Farnsworth B, Guillemin GJ. Age-associated changes in oxidative stress and NAD^+^ metabolism in human tissue. PLoS One. 2012; 7:e42357. 10.1371/journal.pone.0042357 22848760 PMC3407129

[2] Clement J, Wong M, Poljak A, Sachdev P, Braidy N. The plasma NAD^+^ metabolome is dysregulated in “normal” aging. Rejuvenation Res. 2019; 22:121–30. 10.1089/rej.2018.2077 30124109 PMC6482912

[3] Gomes AP, Price NL, Ling AJ, Moslehi JJ, Montgomery MK, Rajman L, White JP, Teodoro JS, Wrann CD, Hubbard BP, Mercken EM, Palmeira CM, de Cabo R, et al. Declining NAD^+^ induces a pseudohypoxic state disrupting nuclear-mitochondrial communication during aging. Cell. 2013; 155:1624–38. 10.1016/j.cell.2013.11.037 24360282 PMC4076149

[4] Belenky P, Racette FG, Bogan KL, McClure JM, Smith JS, Brenner C. Nicotinamide riboside promotes Sir2 silencing and extends lifespan via Nrk and Urh1/Pnp1/Meu1 pathways to NAD^+^. Cell. 2007; 129:473–84. 10.1016/j.cell.2007.03.024 17482543

[5] Yoshino J, Mills KF, Yoon MJ, Imai S. Nicotinamide mononucleotide, a key NAD^+^ intermediate, treats the pathophysiology of diet- and age-induced diabetes in mice. Cell Metab. 2011; 14:528–36. 10.1016/j.cmet.2011.08.014 21982712 PMC3204926

[6] Tchkonia T, Morbeck DE, Von Zglinicki T, Van Deursen J, Lustgarten J, Scrable H, Khosla S, Jensen MD, Kirkland JL. Fat tissue, aging, and cellular senescence. Aging Cell. 2010; 9:667–84. 10.1111/j.1474-9726.2010.00608.x 20701600 PMC2941545

[7] Picard F, Kurtev M, Chung N, Topark-Ngarm A, Senawong T, Machado De Oliveira R, Leid M, McBurney MW, Guarente L. Sirt1 promotes fat mobilization in white adipocytes by repressing PPAR-gamma. Nature. 2004; 429:771–76. 10.1038/nature02583 15175761 PMC2820247

[8] Baur JA, Pearson KJ, Price NL, Jamieson HA, Lerin C, Kalra A, Prabhu VV, Allard JS, Lopez-Lluch G, Lewis K, Pistell PJ, Poosala S, Becker KG, et al. Resveratrol improves health and survival of mice on a high-calorie diet. Nature. 2006; 444:337–42. 10.1038/nature05354 17086191 PMC4990206

[9] Springer M, Moco S. Resveratrol and its human metabolites-effects on metabolic health and obesity. Nutrients. 2019; 11:143. 10.3390/nu11010143 30641865 PMC6357128

[10] Chen H, Liu X, Chen H, Cao J, Zhang L, Hu X, Wang J. Role of SIRT1 and AMPK in mesenchymal stem cells differentiation. Ageing Res Rev. 2014; 13:55–64. 10.1016/j.arr.2013.12.002 24333965

[11] Hardie DG, Schaffer BE, Brunet A. AMPK: an energy-sensing pathway with multiple inputs and outputs. Trends Cell Biol. 2016; 26:190–201. 10.1016/j.tcb.2015.10.013 26616193 PMC5881568

[12] Baas AF, Boudeau J, Sapkota GP, Smit L, Medema R, Morrice NA, Alessi DR, Clevers HC. Activation of the tumour suppressor kinase LKB1 by the STE20-like pseudokinase STRAD. EMBO J. 2003; 22:3062–72. 10.1093/emboj/cdg292 12805220 PMC162144

[13] Alessi DR, Sakamoto K, Bayascas JR. LKB1-dependent signaling pathways. Annu Rev Biochem. 2006; 75:137–63. 10.1146/annurev.biochem.75.103004.142702 16756488

[14] Lan F, Cacicedo JM, Ido Y. Activation of AMPKK-AMPK cascade by Silence Information Regulator 2 (Sir2). Diabetes. 2005; 54:A383.

[15] Lan F, Cacicedo JM, Ruderman N, Ido Y. SIRT1 modulation of the acetylation status, cytosolic localization, and activity of LKB1. Possible role in AMP-activated protein kinase activation. J Biol Chem. 2008; 283:27628–35. 10.1074/jbc.M805711200 18687677 PMC2562073

[16] Lan F, Weikel KA, Cacicedo JM, Ido Y. Resveratrol-induced AMP-activated protein kinase activation is cell-type dependent: lessons from basic research for clinical application. Nutrients. 2017; 9:751. 10.3390/nu9070751 28708087 PMC5537865

[17] Price NL, Gomes AP, Ling AJ, Duarte FV, Martin-Montalvo A, North BJ, Agarwal B, Ye L, Ramadori G, Teodoro JS, Hubbard BP, Varela AT, Davis JG, et al. SIRT1 is required for AMPK activation and the beneficial effects of resveratrol on mitochondrial function. Cell Metab. 2012; 15:675–90. 10.1016/j.cmet.2012.04.003 22560220 PMC3545644

[18] Hou X, Xu S, Maitland-Toolan KA, Sato K, Jiang B, Ido Y, Lan F, Walsh K, Wierzbicki M, Verbeuren TJ, Cohen RA, Zang M. SIRT1 regulates hepatocyte lipid metabolism through activating AMP-activated protein kinase. J Biol Chem. 2008; 283:20015–26. 10.1074/jbc.M802187200 18482975 PMC2459285

[19] Cantó C, Auwerx J. PGC-1alpha, SIRT1 and AMPK, an energy sensing network that controls energy expenditure. Curr Opin Lipidol. 2009; 20:98–105. 10.1097/MOL.0b013e328328d0a4 19276888 PMC3627054

[20] Kirkland JL, Dobson DE. Preadipocyte function and aging: links between age-related changes in cell dynamics and altered fat tissue function. J Am Geriatr Soc. 1997; 45:959–67. 10.1111/j.1532-5415.1997.tb02967.x 9256849

[21] Zoico E, Di Francesco V, Olioso D, Fratta Pasini AM, Sepe A, Bosello O, Cinti S, Cominacini L, Zamboni M. In vitro aging of 3T3-L1 mouse adipocytes leads to altered metabolism and response to inflammation. Biogerontology. 2010; 11:111–22. 10.1007/s10522-009-9236-0 19526322

[22] Alonso I, Baroja A, Fernández B, Vielba R, Elorriaga J, Pérez-Sanz J, Aréchaga J, Goiriena de Gandarias JJ, de la Hoz C. Changes in the expression of cyclin dependent kinase inhibitors during differentiation of immortalized fibroblasts into adipocytes. Int J Dev Biol. 2017; 61:89–93. 10.1387/ijdb.160416cd 28287250

[23] Phelps DE, Xiong Y. Regulation of cyclin-dependent kinase 4 during adipogenesis involves switching of cyclin D subunits and concurrent binding of p18INK4c and p27Kip1. Cell Growth Differ. 1998; 9:595–610. 9716177

[24] Acosta JC, O’Loghlen A, Banito A, Guijarro MV, Augert A, Raguz S, Fumagalli M, Da Costa M, Brown C, Popov N, Takatsu Y, Melamed J, d’Adda di Fagagna F, et al. Chemokine signaling via the CXCR2 receptor reinforces senescence. Cell. 2008; 133:1006–18. 10.1016/j.cell.2008.03.038 18555777

[25] Cacicedo JM, Gauthier MS, Lebrasseur NK, Jasuja R, Ruderman NB, Ido Y. Acute exercise activates AMPK and eNOS in the mouse aorta. Am J Physiol Heart Circ Physiol. 2011; 301:H1255–65. 10.1152/ajpheart.01279.2010 21724864 PMC3197351

[26] Sapkota GP, Kieloch A, Lizcano JM, Lain S, Arthur JS, Williams MR, Morrice N, Deak M, Alessi DR. Phosphorylation of the protein kinase mutated in peutz-jeghers cancer syndrome, LKB1/STK11, at Ser^431^ by p90^RSK^ and cAMP-dependent protein kinase, but not its farnesylation at Cys^433^, is essential for LKB1 to suppress cell vrowth. J Biol Chem. 2001; 276:19469–82. 10.1074/jbc.M009953200 11297520

[27] Tanno M, Sakamoto J, Miura T, Shimamoto K, Horio Y. Nucleocytoplasmic shuttling of the NAD+-dependent histone deacetylase SIRT1. J Biol Chem. 2007; 282:6823–32. 10.1074/jbc.M609554200 17197703

[28] Jing E, Gesta S, Kahn CR. SIRT2 regulates adipocyte differentiation through FoxO1 acetylation/deacetylation. Cell Metab. 2007; 6:105–14. 10.1016/j.cmet.2007.07.003 17681146 PMC2083635

[29] Perrini S, Porro S, Nigro P, Cignarelli A, Caccioppoli C, Genchi VA, Martines G, De Fazio M, Capuano P, Natalicchio A, Laviola L, Giorgino F. Reduced SIRT1 and SIRT2 expression promotes adipogenesis of human visceral adipose stem cells and associates with accumulation of visceral fat in human obesity. Int J Obes (Lond). 2020; 44:307–19. 10.1038/s41366-019-0436-7 31462690

[30] Sinclair DA, Guarente L. Unlocking the secrets of longevity genes. Sci Am. 2006; 294:48–51. 10.1038/scientificamerican0306-48 16502611

[31] Soukas AA, Hao H, Wu L. Metformin as anti-aging therapy: is it for everyone? Trends Endocrinol Metab. 2019; 30:745–55. 10.1016/j.tem.2019.07.015 31405774 PMC6779524

[32] Ido Y, Duranton A, Lan F, Cacicedo JM, Chen TC, Breton L, Ruderman NB. Acute activation of AMP-activated protein kinase prevents H_2_O_2_-induced premature senescence in primary human keratinocytes. PLoS One. 2012; 7:e35092. 10.1371/journal.pone.0035092 22514710 PMC3325987

[33] Ido Y, Duranton A, Lan F, Weikel KA, Breton L, Ruderman NB. Resveratrol prevents oxidative stress-induced senescence and proliferative dysfunction by activating the AMPK-FOXO3 cascade in cultured primary human keratinocytes. PLoS One. 2015; 10:e0115341. 10.1371/journal.pone.0115341 25647160 PMC4315597

[34] Saito Y, Chikenji TS, Matsumura T, Nakano M, Fujimiya M. Exercise enhances skeletal muscle regeneration by promoting senescence in fibro-adipogenic progenitors. Nat Commun. 2020; 11:889. 10.1038/s41467-020-14734-x 32060352 PMC7021787

[35] Ding Y, Chen J, Okon IS, Zou MH, Song P. Absence of AMPKα2 accelerates cellular senescence via p16 induction in mouse embryonic fibroblasts. Int J Biochem Cell Biol. 2016; 71:72–80. 10.1016/j.biocel.2015.12.010 26718972 PMC4720555

[36] Han X, Tai H, Wang X, Wang Z, Zhou J, Wei X, Ding Y, Gong H, Mo C, Zhang J, Qin J, Ma Y, Huang N, et al. AMPK activation protects cells from oxidative stress-induced senescence via autophagic flux restoration and intracellular NAD^+^ elevation. Aging Cell. 2016; 15:416–27. 10.1111/acel.12446 26890602 PMC4854918

[37] Cheng XY, Li YY, Huang C, Li J, Yao HW. AMP-activated protein kinase reduces inflammatory responses and cellular senescence in pulmonary emphysema. Oncotarget. 2017; 8:22513–23. 10.18632/oncotarget.15116 28186975 PMC5410241

[38] Wang W, Yang X, López de Silanes I, Carling D, Gorospe M. Increased AMP:ATP ratio and AMP-activated protein kinase activity during cellular senescence linked to reduced HuR function. J Biol Chem. 2003; 278:27016–23. 10.1074/jbc.M300318200 12730239

[39] Phadke M, Krynetskaia N, Mishra A, Krynetskiy E. Accelerated cellular senescence phenotype of GAPDH-depleted human lung carcinoma cells. Biochem Biophys Res Commun. 2011; 411:409–15. 10.1016/j.bbrc.2011.06.165 21749859 PMC3154080

[40] Sung JY, Woo CH, Kang YJ, Lee KY, Choi HC. AMPK induces vascular smooth muscle cell senescence via LKB1 dependent pathway. Biochem Biophys Res Commun. 2011; 413:143–48. 10.1016/j.bbrc.2011.08.071 21872575

[41] Yi G, He Z, Zhou X, Xian L, Yuan T, Jia X, Hong J, He L, Liu J. Low concentration of metformin induces a p53-dependent senescence in hepatoma cells via activation of the AMPK pathway. Int J Oncol. 2013; 43:1503–10. 10.3892/ijo.2013.2077 23982736

[42] Lanna A, Henson SM, Escors D, Akbar AN. The kinase p38 activated by the metabolic regulator AMPK and scaffold TAB1 drives the senescence of human T cells. Nat Immunol. 2014; 15:965–72. 10.1038/ni.2981 25151490 PMC4190666

[43] Liao EC, Hsu YT, Chuah QY, Lee YJ, Hu JY, Huang TC, Yang PM, Chiu SJ. Radiation induces senescence and a bystander effect through metabolic alterations. Cell Death Dis. 2014; 5:e1255. 10.1038/cddis.2014.220 24853433 PMC4047910

[44] Imamura K, Ogura T, Kishimoto A, Kaminishi M, Esumi H. Cell cycle regulation via p53 phosphorylation by a 5'-AMP activated protein kinase activator, 5-aminoimidazole- 4-carboxamide-1-beta-D-ribofuranoside, in a human hepatocellular carcinoma cell line. Biochem Biophys Res Commun. 2001; 287:562–67. 10.1006/bbrc.2001.5627 11554766

[45] Jones RG, Plas DR, Kubek S, Buzzai M, Mu J, Xu Y, Birnbaum MJ, Thompson CB. AMP-activated protein kinase induces a p53-dependent metabolic checkpoint. Mol Cell. 2005; 18:283–93. 10.1016/j.molcel.2005.03.027 15866171

[46] Fogarty S, Ross FA, Vara Ciruelos D, Gray A, Gowans GJ, Hardie DG. AMPK causes cell cycle arrest in LKB1-deficient cells via activation of CAMKK2. Mol Cancer Res. 2016; 14:683–95. 10.1158/1541-7786.MCR-15-0479 27141100 PMC5390849

[47] Lee JH, Jang H, Lee SM, Lee JE, Choi J, Kim TW, Cho EJ, Youn HD. ATP-citrate lyase regulates cellular senescence via an AMPK- and p53-dependent pathway. FEBS J. 2015; 282:361–71. 10.1111/febs.13139 25367309

[48] Li P, Zhao M, Parris AB, Feng X, Yang X. P53 is required for metformin-induced growth inhibition, senescence and apoptosis in breast cancer cells. Biochem Biophys Res Commun. 2015; 464:1267–74. 10.1016/j.bbrc.2015.07.117 26225749

[49] Cantó C, Gerhart-Hines Z, Feige JN, Lagouge M, Noriega L, Milne JC, Elliott PJ, Puigserver P, Auwerx J. AMPK regulates energy expenditure by modulating NAD+ metabolism and SIRT1 activity. Nature. 2009; 458:1056–60. 10.1038/nature07813 19262508 PMC3616311

[50] Devin A, Guérin B, Rigoulet M. Cytosolic NAD^+^ content strictly depends on ATP concentration in isolated liver cells. FEBS Lett. 1997; 410:329–32. 10.1016/s0014-5793(97)00612-1 9237656

[51] Hardie DG. Biochemistry. Balancing cellular energy. Science. 2007; 315:1671–72. 10.1126/science.1140737 17379794

[52] Beirowski B, Babetto E, Golden JP, Chen YJ, Yang K, Gross RW, Patti GJ, Milbrandt J. Metabolic regulator LKB1 is crucial for schwann cell-mediated axon maintenance. Nat Neurosci. 2014; 17:1351–61. 10.1038/nn.3809 25195104 PMC4494117

[53] Gan B, Hu J, Jiang S, Liu Y, Sahin E, Zhuang L, Fletcher-Sananikone E, Colla S, Wang YA, Chin L,. Depinho RA Lkb1 regulates quiescence and metabolic homeostasis of haematopoietic stem cells. Nature. 2010; 468:701–04. 10.1038/nature09595 21124456 PMC3058342

[54] Gurumurthy S, Xie SZ, Alagesan B, Kim J, Yusuf RZ, Saez B, Tzatsos A, Ozsolak F, Milos P, Ferrari F, Park PJ, Shirihai OS, Scadden DT, Bardeesy N. The Lkb1 metabolic sensor maintains haematopoietic stem cell survival. Nature. 2010; 468:659–63. 10.1038/nature09572 21124451 PMC3037591

[55] Nakada D, Saunders TL, Morrison SJ. Lkb1 regulates cell cycle and energy metabolism in haematopoietic stem cells. Nature. 2010; 468:653–58. 10.1038/nature09571 21124450 PMC3059717

[56] Gormand A, Henriksson E, Ström K, Jensen TE, Sakamoto K, Göransson O. Regulation of AMP-activated protein kinase by LKB1 and CaMKK in adipocytes. J Cell Biochem. 2011; 112:1364–75. 10.1002/jcb.23053 21312243

[57] Hallstrom TC, Mori S, Nevins JR. An E2F1-dependent gene expression program that determines the balance between proliferation and cell death. Cancer Cell. 2008; 13:11–22. 10.1016/j.ccr.2007.11.031 18167336 PMC2243238

[58] Zebisch K, Voigt V, Wabitsch M, Brandsch M. Protocol for effective differentiation of 3T3-L1 cells to adipocytes. Anal Biochem. 2012; 425:88–90. 10.1016/j.ab.2012.03.005 22425542

[59] Ido Y, Carling D, Ruderman N. Hyperglycemia-induced apoptosis in human umbilical vein endothelial cells: inhibition by the AMP-activated protein kinase activation. Diabetes. 2002; 51:159–67. 10.2337/diabetes.51.1.159 11756336

[60] Houde VP, Ritorto MS, Gourlay R, Varghese J, Davies P, Shpiro N, Sakamoto K, Alessi DR. Investigation of LKB1 Ser431 phosphorylation and Cys433 farnesylation using mouse knockin analysis reveals an unexpected role of prenylation in regulating AMPK activity. Biochem J. 2014; 458:41–56. 10.1042/BJ20131324 24295069 PMC3898322

